# Ageing well: evaluation of social participation and quality of life tools to enhance community aged care (study protocol)

**DOI:** 10.1186/s12877-019-1094-2

**Published:** 2019-03-12

**Authors:** Lindsey Brett, Andrew Georgiou, Mikaela Jorgensen, Joyce Siette, Grace Scott, Edwina Gow, Gemma Luckett, Johanna Westbrook

**Affiliations:** 1 Centre for Health Systems and Safety Research, Australian Institute of Health Innovation, Macquarie University, Level 6, 75 Talavera Road, Sydney, NSW 2109 Australia; 2 School of Psychology, The University of Queensland, St Lucia, QLD 4072 Australia; 3 Uniting, 2 Chapman Avenue, Chatswood, NSW 2067 Australia; 4 Centre for Research Innovation and Advocacy, Uniting, 13 Blackwood Place, North Parramatta, NSW 2151 Australia; 5 Department of Health Professions, Faculty of Medicine and Health Sciences, Macquarie University, Ground Level, 75 Talavera Raod, Sydney, NSW 2109 Australia

**Keywords:** Social participation, Quality of life, Home care, Aged community care

## Abstract

**Background:**

Several outcome measures can be utilised to measure social participation and Quality of Life (QoL) in research and clinical practice. However there have been few large-scale trials of these tools in community care to identify their value to clients and providers. This study aims to evaluate the implementation of the Australian Community Participation Questionnaire (ACPQ) and the ICEpop CAPability measure for Older people (ICECAP-O) as tools to measure social participation and QoL for clients receiving community aged care services. The specific research questions focus on determining: (1) the levels and predictors of social participation and QoL among older adults using community aged care services; (2) the acceptability and feasibility of implementation of ACPQ and ICECAP-O tools into routine community aged care assessments; (3) if implementation of the tools change service provision and outcomes for older adults receiving community aged care services.

**Methods:**

A mixed method design will be used to collect data from a large Australian aged care provider. Community aged care clients’ ACPQ and ICECAP-O scores, as well as other key outcomes (e.g. services used, hospitalisation and admission to permanent residential care), will be examined at baseline and 12-monthly follow-up assessments. Interviews and focus groups with community aged care clients and staff who administer the tools will also be completed. Descriptive statistics and multiple linear regression will be used to examine the levels and predictors of social participation and QoL. Thematic analysis of interviews and focus groups will be used to determine the acceptability and feasibility of implementing the ACPQ and ICECAP-O into routine needs assessments in community aged care. Case-controlled analyses will be used to determine whether the implementation of the ACPQ and ICECAP-O changes service use and outcomes.

**Discussion:**

The novel use of the ACPQ and the ICECAP-O tools as part of routine needs assessments for community aged care clients has the potential to improve the quality and effectiveness of community aged care services and outcomes.

**Trial registration:**

Australian and New Zealand clinical trial registry number: ACTRN12617001212347. Registered 18/08/2017

**Electronic supplementary material:**

The online version of this article (10.1186/s12877-019-1094-2) contains supplementary material, which is available to authorized users.

## Background

Community aged care providers face numerous challenges in meeting the growing needs of older adults, and catering for their transition across health and social services. One challenge, often overlooked, is the risk of loneliness and social isolation. Approximately a quarter of older Australians live alone in a private dwelling [1] and evidence suggests that up to one third or more older adults will experience some degree of loneliness [2]. Older adults are at risk of feeling a lack of companionship or meaningful connections with others due to a number of factors associated with ageing [3]. Risk factors include the absence of a partner or children, and living in a rural or remote location [2]; health indicators such as dementia, obesity, physical disabilities and depression [4]; and life events, such as a loss of partner or bereavement [3].

Many older adults express a desire to remain living in their own homes for as long as possible, and to stay connected as contributing members of their communities [5]. Targeted community aged care services are a central way of supporting older adults to achieve their health goals, enabling them to be more independent within their own homes and the community. However, community aged care service provision has tended to focus on meeting specific physical needs of older adults rather than targeting strategies which may be effective in improving their social participation and reducing loneliness [6]. International studies have demonstrated that consideration of psychosocial needs, such as participation in meaningful activities, as part of community aged care assessment and service provision can enhance choice, improve quality of life (QoL) and reduce carer burden [7, 8]. High levels of social participation among community-dwelling older adults are associated with lower levels of psychological distress [9–17], greater happiness and satisfaction with life [10], higher self-rated health [18, 19], better physical function [20], lower risk of future dependence for Activities of Daily Living (ADLs) [21], and reduced mortality [22]. Social participation also generates societal benefits through older adults volunteering and community contributions to neighbourhood associations, religious groups or non-governmental organisations [21].

Several tools have been developed to measure social participation and QoL, including some that have been specifically designed or adapted for older populations. There are comprehensive assessment tools, such as the Older American Resources and Services (OARS) assessment and the LEIPAD questionnaire, which consider social well-being and QoL along with other aspects of general health [23]. There are also tools which focus solely on social participation or QoL, such as the Australian Community Participation Questionnaire (ACPQ) [13], Adult Social Care Outcomes Toolkit (ASCOT) [24], ICEpop CAPability measure for Older people (ICECAP-O) [25] and the Medical Outcomes Study Questionnaire Short Form (SF-36) [26]. When measuring social participation and QoL in both research and clinical practice it is important to select tools that are valid, reliable, feasible, and economical [27]. The setting in which these tools are used also influences the selection process; tools used in research can be very detailed to address specific research aims, while in clinical practice tools are selected dependent on the goals of care and often need to be quick to complete and suitable for a wide population [27].

A review identified the ACPQ as a valid tool (good construct validity and reasonable concurrent validity) for assessing social participation in the general population of Australia [28], and the ICECAP-O as a reliable measure of wellbeing and QoL for older adults [29, 30]. The findings of the systematic reviews and consultation with key stakeholders from a large Australian aged care provider led to a feasibility study examining the integration of the ACPQ and ICECAP-O tools into community aged care needs assessments [31]. The aims of the feasibility study were to determine the acceptability of the tools to help plan, design and monitor social participation services in the community aged care setting. More than 300 older adults and 12 community aged care staff were involved. Feedback from the staff was very positive, and many felt the tools would provide information that could help to identify services to support clients’ needs [31, 32]. The feasibility study demonstrated the potential value of implementing this approach on a larger scale and informed the design of this current study [33].

### Aim and research questions

Our aim is to evaluate the implementation of the ACPQ and the ICECAP-O as tools to measure social participation and QoL in clients receiving community aged care services.

Our specific research questions are:

1. What are the levels and predictors of social participation and QoL among older adults using community aged care services (including associations between social participation and QoL)?
2. Is the implementation of ACPQ and ICECAP-O tools into routine needs assessment acceptable and feasible for clients and staff?
3. Does the implementation of the tools affect the volume of services provided and outcomes (e.g. admission to permanent residential care) for older adults receiving community aged care services?

## Methods

A mixed method design will be adopted with the aim of evaluating the implementation of the ACPQ and ICECAP-O as part of routine needs assessments by one of Australia’s largest community aged care providers. Quantitative and qualitative data will be collected over an 18-month period (data collection will cease July 2019) to gain an understanding of the levels and predictors of social participation and QoL for older adults that use community aged care services, and the impact and acceptability of the ACPQ and ICECAP-O on community aged care client, staff and service provision. The study will be implemented in three iterative waves within New South Wales (NSW) and the Australian Capital Territory (ACT), Australia. For this study a wave is defined as a stage of the study when data will be collected (quantitative data from routine needs assessments and qualitative data from interviews and focus groups) from a predetermined region. During this time the research team will obtain regular feedback from community aged care clients and staff on the implementation process, as a means of continuing to adjust and improve the implementation process for each successive implementation wave.

Ethical approval was granted by the Macquarie University Human Research Ethics Committee (reference number: 5201700912). The study has been registered with the Australian and New Zealand Clinical Trials Registry (trial ID: ACTRN12617001212347) [34]. Quantitative data will be reported as per the REporting of studies Conducted using Observational Routinely-collected health Data (RECORD) statement [35]. The COnsolidated criteria for REporting Qualitative research (COREQ): a 32-item checklist for interviews and focus groups, will be utilised for qualitative data [36]. Informed written consent will be provided by all participants (or a proxy as required) who agree to take part in interviews and focus groups. Quantitative data provided by the aged care provider via a secure platform will be nonidentifiable.

### Participants and setting

This is a collaborative study between researchers at Macquarie University and Uniting, one of the largest community aged care providers in NSW and the ACT. Uniting community aged care helps older adults access various services, such as gardening and light housework, meal preparation, shopping, day and overnight respite care, social support (individual or group), nursing and personal care, medication, rehabilitation, and exercise physiology [37]. The study will be conducted across a selection of Uniting community aged care regions within NSW and the ACT. New South Wales is located on the south-east coast of Australia and is home to over one third of Australia’s population with over 7.7 million people [38]. The ACT is a small federal district (population of 403,468) housing the country’s capital, Canberra [38]. Both NSW and the ACT populations are highly urbanised and ethnically diverse. Other than Australia, residents of NSW and the ACT most commonly originate from China, England, India, New Zealand and Philippines [39].

#### Community aged care clients

Uniting will provide nonidentifiable data for all community aged care clients that are living in NSW and the ACT during the study period, including those who have completed the ACPQ and ICECAP-O as part of a routine needs assessment. This will enable a case-controlled study design to be implemented. In 2016, Uniting provided community aged care services to over 6800 clients aged 65 years and older.

During completion of the ACPQ and ICECAP-O, approximately 30 community aged care clients will be invited by Uniting staff to participate in interviews and focus groups to help the research team to gain insights into the feasibility and acceptability of the ACPQ and ICECAP-O (research question two), and how they have affected service use (research question three). Community aged care staff will identify and invite community aged care clients with varying levels of independence, care needs, ability to communicate, and from culturally and linguistically diverse backgrounds that have the ability to provide consent (or proxy consent) and answer questions to participate in an interview or focus group setting.

#### Community aged care staff

Prior to data collection, the research team will provide all community aged care staff that complete needs assessments within the three study regions with training in the administration and purpose of the ACPQ and ICECAP-O. Community aged care staff will then complete the ACPQ and ICECAP-O with all their clients during routine needs assessments. Approximately 30 staff will be invited to participate in interviews and focus groups during training and feedback sessions with the research team to gain an insight into their perspectives regarding acceptability of the ACPQ and ICECAP-O, their experiences in using the tools (research question two), and the perceived impact the tools have on client discussions and service provision (research question three).

### Instruments

#### ACPQ

**Fig. 1:**
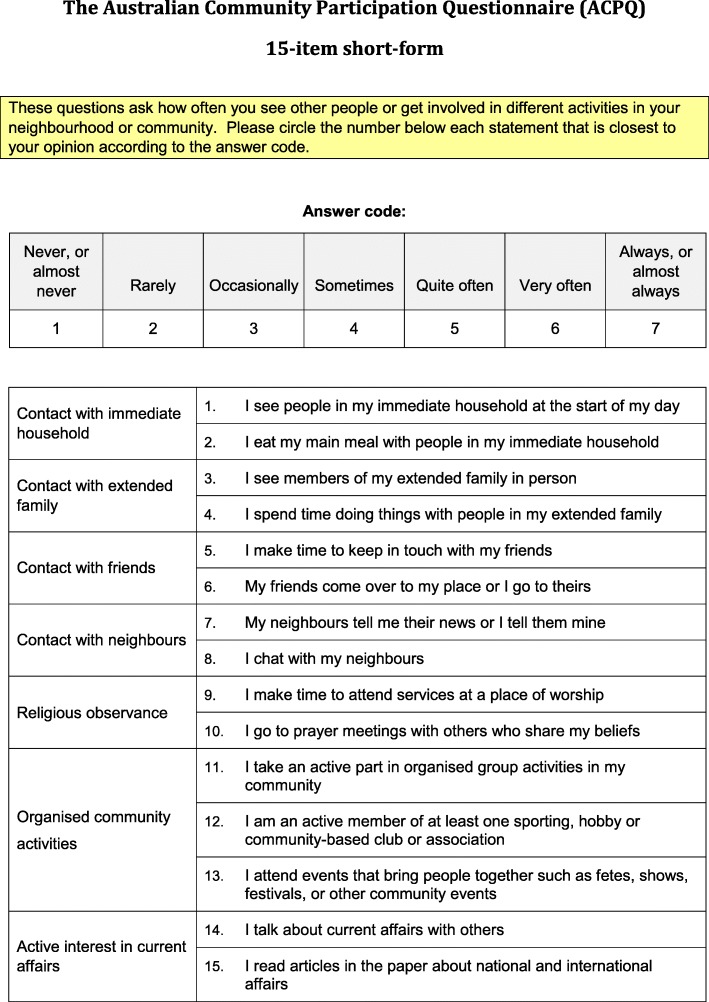
The Australian Community Participation Questionnaire (ACPQ) 15-item short-form

The ACPQ was developed to measure social participation and has been tested in Australia with older adults (Fig. 1). The 15-item version of the ACPQ will be used in this study [40]. The ACPQ taps into seven separate types of participation: contact with immediate household; contact with extended family; contact with friends; contact with neighbours; religious observance; organised community activities; and active interest in current affairs [13]. Participants respond to each item using a seven-point Likert scale ranging from “never, or almost never” (1) to “always, or almost always” (7). An index of breadth of participation can be derived by dichotomising average scores for each participation type using a mean-split as follows: a score of one indicates a ‘participator’ for those at or above the mean, or zero for ‘non-participators’, i.e. those below the mean. This mean-split procedure is conducted for each of the seven types [13]. Scores are then summed to give an eight-point index with a range of zero to seven.

#### ICECAP-O

The ICECAP-O is a measure of capability and will be used in this study to assess QoL (available from https://www.birmingham.ac.uk/research/activity/mds/projects/HaPS/HE/ICECAP/ICECAP-O/index.aspx). This tool was developed in the United Kingdom (UK) and has been used in Australian studies [41–43]. The ICECAP-O assesses wellbeing and QoL using a five-item multiple choice questionnaire according to five attributes: attachment (love and friendship), security (thinking about the future without concern), role (doing things that make you feel valued), enjoyment (enjoyment and pleasure) and control (independence) [25]. Each domain has four possible response options. The ICECAP-O can be used to calculate a global capability index score on a zero to one scale where zero represents no capability and one represents full capability [25].

### Evaluation

Interviews and focus groups will be conducted with community aged care clients and staff throughout the study by experienced researchers (Additional file 1 outlines interview and focus group questions). They will be conducted either at Uniting Centres, the participants’ own homes or via telephone, dependent on what is most suitable for each participant. Audio-recording of the interviews and focus groups will be completed to ensure accuracy of the information gathered. Qualitative data collected from interviews and focus groups analysis will be managed using NVivo 12 software. All quantitative analyses will be performed using SAS 9.4 or StataMP 15.

#### Research question one: What are the levels and predictors of social participation and QoL among older adults using community aged care services (including associations between social participation and QoL)?

##### Variable and measures

Non-identifiable demographic and service data on clients receiving community care services will be extracted monthly by Uniting from their care management system (CareLink+) and made available to researchers through a secure file sharing platform during the study period. Clients’ demographics (e.g. year of birth, gender), care needs, service use, ACPQ and ICECAP-O data, hospitalisations, admission to permanent residential care, mortality and other outcomes as specified in Additional file 2 will be extracted.

##### Data analyses

The study population for this part of the analysis will include all older adults receiving community aged care services who are administered the social participation and QoL tools over the study period (estimated minimum sample size required: 720–961). Descriptive statistics will be used to quantify the types, breadth and levels of social participation and QoL at baseline and changes in social participation and QoL at 12-monthly follow-up assessments. Multiple linear regression will be used to examine the association between social participation and QoL at baseline. Other possible explanatory variables will include socio-demographic variables listed in Additional file 2.

#### Research question two: Is the implementation of ACPQ and ICECAP-O tools into routine needs assessment acceptable and feasible for older adults and staff?

##### Variable and measures

Interviews and focus group will be conducted with a purposive sample of Uniting community aged care clients (estimated *n* = 30) and staff (estimated n = 30) to generate context-rich data to determine if implementation of the ACPQ and ICECAP-O was considered feasible and acceptable. Examples of questions that will be used include ‘was there anything you found difficult or unpleasant?’ (client interviews and focus groups), and ‘did you find the information that you gathered from this useful and valuable? In what ways?’ (staff interviews and focus groups) (Additional file 1).

##### Data analyses

Interviews and focus groups will be recorded and transcribed verbatim. One researcher will systematically perform the initial open coding of the data, which will then be reviewed and refined by the research team. Any discrepancies in coding will be discussed and used to modify the list of codes until all researchers agree on code application. The codes will then be sorted into preliminary domains and themes, which will be repeatedly reviewed and refined by the research team to maximise homogeneity prior to developing an analytic narrative.

#### Research question three: Does the implementation of the tools affect service provision and outcomes for older adults receiving community aged care services?

##### Variable and measures

Actions taken by staff and/or clients following the assessments will be identified by collection of the data inputted into the ‘additional comments’ free text box located in the ACPQ and ICECAP-O electronic forms. Staff may identify if there was an addition of a new service, an existing service was changed or if there were no changes. All Uniting services utilised by clients are also captured within the Carelink+ care management system by start time, end time, date and type of service. These data will be extracted along with all other non-identifiable data required to answer research questions one and three.

Interviews and focus group (as outlined above) will also be used to determine if the implementation of the ACPQ and ICECAP-O influenced service provision. Examples of interview and focus group questions specific to research question three include ‘do you think that answering and discussing these questions with your support advisor led to any changes in your services? Can you give any examples?’ (client questions), and ‘did the information you gathered affect your care planning? Can you give any examples?’ (staff questions) (Additional file 1).

##### Data analyses

The first part of the analysis for this research question aims to determine whether the actions identified by staff following administration of the tools result in a change in social participation and QoL scores at 12-monthly follow-up needs assessments. Individual growth modelling will be used to examine changes over time in social participation and QoL scores for all older adults who are administered the tools at two or more-time points.

The second part of the analysis will use case-controlled analyses to determine whether the implementation of the tools increases the volume of social support service use and improves outcomes. A control group of clients who do not receive the tools will be identified from the data on all community care clients extracted by Uniting. Each person who receives the tools will be matched to a person who does not receive the tools, based on their sociodemographic and service use characteristics at the time of their routine reassessment. Propensity score matching methods will be used to reduce the impact of confounding and selection bias that can occur in observational studies [44]. Volume and frequency of services following routine assessment will be compared between case and control groups using general linear modelling. Time to entry into residential aged care and frequency of adverse events (e.g. hospitalisations) will be compared between case and control groups using competing risks regression and negative binomial regression, respectively.

Interview and focus group data will be analysed as outlined earlier in Research Question Two.

### Sample size and power calculation

A total sample size of between 720 and 961 older adults is needed to compare volume of social support service use between clients using the ACPQ/ICECAP-O assessment tools (cases) and a matched sample of those who are not assessed (controls) using general linear modelling (80 and 90% power, respectively). This sample size estimate assumes an R-squared value of 0.1 for the full model and inclusion of up to 10 covariates. A sample size of between 830 and 1110 clients is needed in each of the case and control groups to detect a 15% risk reduction in entry into residential aged care with a 12-month follow-up period (80 and 90% power, respectively).

For the interviews and focus groups, it is estimated that a sample size of 30 community aged care clients and 30 staff will achieve data saturation (i.e. no new themes are emerging). These estimations are based on earlier studies conducted by the research team in this area [31].

## Discussion

This paper has described the methods that will be utilised to evaluate the implementation of social participation and QoL assessment tools (ACPQ and ICECAP-O) in community aged care. It is anticipated that this intervention will enhance the provision of targeted services, and thus increase social participation and QoL for older adults living in the community setting. By using a mixed method approach, this study allows for the collection of qualitative data that provides rich insights into community aged care clients’ and staff experiences. Adopting ACPQ and ICECAP-O tools into routine needs assessments for community aged care clients is a potentially simple and effective way to gain understanding into older adults’ levels of social participation and QoL. Community aged care staff can use this information to discuss future strategies to increase social participation and QoL with their clients. This project will also provide the opportunity to consider the validity and reliability of the ACPQ and ICECAP-O among community aged care clients in Australia.

### Strategies to increase validity and reduce bias

By using an iterative, multi-method design for this study, the different approaches will be used as a form of validation of the implementation process used. The methods that will be utilised to address the study objectives include the collection and analysis of quantitative data from the CareLink+ database, interviews and focus groups to understand staff and client perceptions of social participation and the implementation of the tools, and adaptation of the implementation process at each wave based on staff feedback during training and feedback sessions. The use of methodological triangulation will help to reduce bias and deficiencies associated with using a single method design [45]. Steps will be taken to check the representation of older Australians within the study sample through comparison of demographic data from the study population and older adults living within NSW and the ACT.

Uniting community aged care staff will be provided with training and feedback by the research team on how to administer the ACPQ and the ICECAP-O tools prior to implementing the tools with clients. The training will include information on how to access the forms via the Uniting care management system (CareLink+) and how to ask the questions effectively. During each wave the Working Group Committee will review the data and forward any issues and resolutions to staff as required during the succeeding waves.

### Limitations

This study will use an iterative approach to allow for review and changes to the implementation process across the three waves. This approach will allow for greater understanding of the data and improvements that will help to strengthen the implementation process over time. One limitation of this approach is the inability to control for all factors due to changes made at each wave. However, the aim of this study is to evaluate the implementation of the ACPQ and ICECAP-O so the ability to be able to review and adjust this process is required. The quantitative analyses will also account for the clustered nature of the data within each wave.

Focus groups and interviews with Uniting staff and clients will produce indicative and rich data about social participation and the implementation of the ACPQ and ICECAP-O. All staff and clients that complete both tools will be invited to participate in this process to help ensure a representative sample is used. However, participation in this element of the study is voluntary and the data collected are based on the perceptions of the participants, which could potentially limit the generalisability of the findings from this study.

Similar to other studies conducted with older adults, there is a potential limitation associated with sample size and high attrition rates. Relocation to residential aged care and mortality are attrition risks in research involving community aged care clients. However, this study will be undertaken in partnership with one of the largest aged care providers in Australia. Targeting both new and existing clients across Uniting’s large client population will allow for statistical power to be optimised, as well as the potential to explore the impact of the intervention on specific vulnerable groups.

## Additional files

Additional file 1: Community aged care client and coordinator interview/focus group questions (List of questions to be asked during interviews and focus groups with community aged care clients and staff) (DOCX 16 kb)

Additional file 2: Sociodemographic, service provision and outcome variables from Uniting data systems (List of variables that will be extracted from the data systems for analysis as part of this study) (DOCX 14 kb)

## Abbreviations

ACPQ: Australian Community Participation Questionnaire
ACT: Australian Capital Territory
ADLs: Activities of Daily Living
COREQ: COnsolidated criteria for REporting Qualitative research
ICECAP-O: ICEpop CAPability Measure for Older Adults
NSW: New South Wales
QoL: Quality of Life
RECORD: Reporting of studies Conducted using Observational Routinely-collected health Data
