# Supplementary material for: Ageing well: evaluation of social participation and quality of life tools to enhance community aged care (study protocol)
Source: BMC Geriatr. 2019 Mar 12;19:78. doi: 10.1186/s12877-019-1094-2 (PMC6419453; doi:10.1186/s12877-019-1094-2)
Supplement: Supplementary file 1 — Community aged care client and coordinator interview/focus group questions (List of questions to be asked during interviews and focus groups with community aged care clients and staff) (DOCX 16 kb) [file 12877_2019_1094_MOESM1_ESM.docx]

***For aged care clients:***

1. What was it like completing the questionnaires/surveys? How was the experience for you?

2. Was there anything that you found particularly helpful?

3. Was there anything you found difficult or unpleasant?

4. Overall, do you think completing these questionnaires/surveys with your support advisor was useful? In what ways?

5. Do you think that answering and discussing these questions with your support advisor have led to any changes in your services? Can you give any examples?

6. How have the questionnaires/surveys, or the services you received impacted on your social life and your quality of life?

7. How has your social life changed since coming to Uniting?

8. Have you or someone you know experienced the feeling of loneliness? Can you give an example?

9. Do you think participating in activities like the services provided by Uniting helps to address the loneliness you feel? If yes, can you explain how?

10. Do you think the questionnaires/surveys can help to address loneliness and/or encourage socialising?

11. What activities do you find help you to feel less lonely and encourage socialising with others?

***For aged care staff:***

1. What was it like administering these questionnaires? How was the experience for you?

2. What were your clients’ reactions to the tools?

3. Did you experience any difficulties administering the tools? …Were any particular questions more difficult to ask than others?

4. Did you find the information that you gathered from this useful and valuable? In what ways?

5. Did the information you gathered affect your care planning? Can you give any examples?

6. How do you feel about the support you were given, in terms of training? Is there anything else you think would have been helpful?

7. Do you think that discussing these questions with your client have led to any changes in the services provided to individual clients? Can you give any examples of where the information gained influenced services for clients?
