# Supplementary material for: Ageing well: evaluation of social participation and quality of life tools to enhance community aged care (study protocol)
Source: BMC Geriatr. 2019 Mar 12;19:78. doi: 10.1186/s12877-019-1094-2 (PMC6419453; doi:10.1186/s12877-019-1094-2)
Supplement: Supplementary file 2 — Sociodemographic, service provision and outcome variables from Uniting data systems (List of variables that will be extracted from the data systems for analysis as part of this study) (DOCX 14 kb) [file 12877_2019_1094_MOESM2_ESM.docx]

**Appendix 1:** Sociodemographic, service provision and outcome variables from Uniting data systems^1^

| *Sociodemographics* | *Details* |
| --- | --- |
| Age | Year of birth only |
| Gender | Male, female, not stated |
| Marital status | Married, defacto, divorced, single, widowed |
| Pension status | Type of pension e.g. age pension, disability support pension |
| Carer status | Client has a carer (yes/no) |
| Country of birth | ABS Standard Australian Classification of Countries 2011 |
| Language spoken | Language spoken by the client |
| Geographic location | Suburb and postcode of residence; Link to area-level remoteness (ARIA+) and socioeconomic status (IRSAD) using the ABS Australian Statistical Geography Standard 2011 |
| Care needs | Activities of Daily Living: dressing, eating, toileting, walking, bathing; Instrumental Activities of Daily Living: housework, getting places, shopping, taking medicine, handling money |
| *Service provision* |  |
| Care program | Care program, time to change in program/level |
| Services used | Service types, frequency of services, volume of services |
| *Outcomes* |  |
| Social participation | Australian Community Participation Questionnaire (ACPQ) |
| Quality of life | ICEpop CAPability measure for Older people (ICECAP-O) |
| Mortality | Time to death |
| Hospitalisations | Frequency of hospital leave, time in hospital |
| Admission to permanent residential care | Time to entering residential aged care facility |

^1^ Additional data field(s) for care planning may be extracted or co-developed
